# Environmental Radiation Monitoring at NBS/NIST From 1960 Through 2000

**DOI:** 10.6028/jres.106.037

**Published:** 2001-10-01

**Authors:** Thomas G. Hobbs

**Affiliations:** National Institute of Standards and Technology, Gaithersburg, MD 20899-3541

**Keywords:** electret detectors, environment, G-M detectors, ion chamber detectors, radiation, radioactivity, scintillation detectors, thermoluminescent detectors

## Abstract

The program for monitoring the environment in and about the site of the National Bureau of Standards, now the National Institute of Standards and Technology, at its Gaithersburg, Maryland location began in 1960. The program includes measurements of radiation fields at the fence line of the site and of radionuclides in samples of soil, water, and biota taken within and around the site. A variety of instruments and equipment, processes and procedures, and measurement devices has been employed. To date, no measurement from the routine program has exhibited any result that could be attributed to any effluent or other effect of the radiological work conducted at the site; that includes the NIST Research Reactor, the now defunct Linear Electron Accelerator (LINAC) and other accelerators, radiochemistry, and sealed source operations.

## 1. Introduction

In about mid-1960, Dr. A. Schwebel and Tom Hobbs, the two staff Health Physicists at the National Bureau of Standards (NBS), now the National Institute of Standards and Technology (NIST), drove to Gaithersburg, Maryland from the National Bureau of Standards in Washington, DC. They brought with them a portable radiation survey meter in order to survey the new location that had been chosen for the National Bureau of Standards. Believing they were surveying the new location for the agency, they unfortunately chose to walk through the field at the northeast quadrant of the I-70S (now I-270) overpass at Diamond Avenue, thinking that dirt piles from the interstate construction in that field were from construction for the new NBS site. In fact, the new site for the National Bureau of Standards was to be in the field at the opposite corner. Surveys of the correct area had to await another day. This somewhat farcical test proved a most inauspicious initiation into environmental monitoring.

Environmental monitoring at the Washington, DC site had consisted of process-specific surveys using the available technology of the time. Survey meters with a variety of detectors were turned on and observed during the frequent and wide ranging walk-around tours of the facility and trips between buildings; laboratory instruments such as ion chambers and scintillation spectrometers were occasionally set up outside buildings; high volume air samplers were employed for analytical measures of air quality during specific laboratory processes; and radiochemical analyses of samples of soil and other materials, e.g., pavement and leaves, were employed as spot tests of radioactive material control effectiveness.

Shortly after the announcement that the facility would move to a new location, and the selection of the location, NBS Health Physics began planning a program of routine monitoring for radiation effluents in the new NBS environment that continues to the present. Throughout the years preceding and following the first occupancy of the Gaithersburg site [1,2], many and varied instruments and equipment systems, and processes and procedures have been employed to assess the quality and the quantity of the radiations in the environment of the NBS/NIST Gaithersburg site and its surrounding vicinity. In addition to measurements of penetrating external radiation fields with integrating and with real-time devices on the fence line, the routine program includes collection, processing, measurement, and analysis of samples of grass, soil, and water from the NIST environment. The results of the routine programs of monitoring and sampling have demonstrated that no measurable effluents have been observed that could be attributed to NBS/NIST radiological programs.

The radiochemical operations that could have contributed effluents to the environment began shortly after the laboratories were occupied in 1965. Local controls on the fume hoods, with absolute or high efficiency particulate air (HEPA) filters installed as necessary for a particular operation, ensured that no measurable releases to the environment have occurred, as demonstrated with local monitoring at the potential release point.

The linear electron accelerator (LINAC) began operations with full beam production and handling in 1965. The beam level, in the subbasement of Bldg. 245, Radiation Physics, was ventilated with a one-pass system that exhausted approximately 12 000 L/s (25 000 cfm) through the 30.5 m (100 ft) stack at the south side of the building. Primary contaminants expected, and the only ones ever detected, were the short-lived gaseous ^13^N and ^15^O, from the (n,2n) reactions with natural nuclides in air. A gamma detector was placed at the base of the stack and measurements were made from the start of operations until the LINAC was deactivated in 1987. From that data and from calculations using the dilution that would occur with the stack height and the high volume of air exhausted, and from measurements made in the vicinity of the stack outside the building, it was determined that no measurable effluents reached the environment.

The Research Reactor (NBSR) went critical in December of 1967, reached 10 MW early in 1969, and went to 20 MW in 1995. Building 235, Reactor, has a stack with height and exhaust rate equivalent to the Bldg. 245 stack. Monitors have been mounted inside the stack throughout the life of the facility. Measurements with those and with devices outside the building, and calculations with accepted dilution factors for exhaust have indicated that no significant impact on the environment has resulted from reactor operations.

**Fig. 1:**
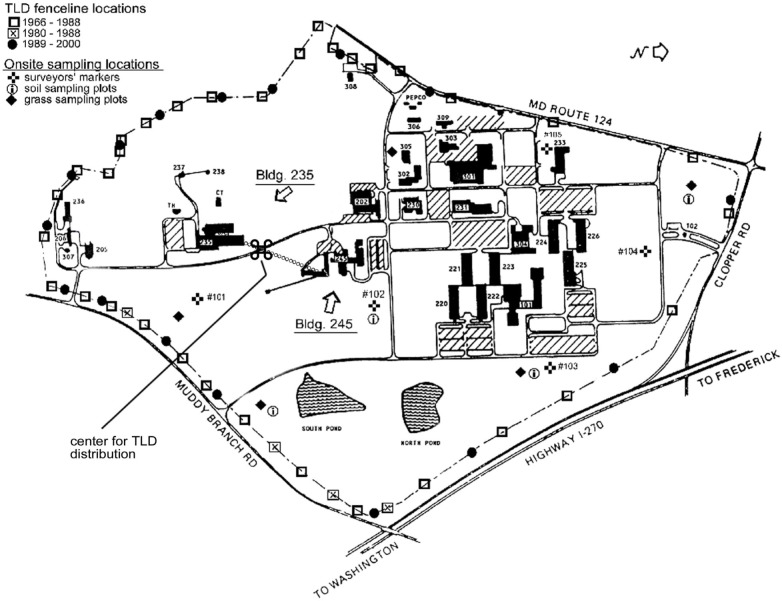
A representation of the NIST site, showing locations for the various monitor and sampling stations on and within the fence line. The center point for quadrant grouping locations is indicated. Offsite monitors have been as close as three miles away and as far away as thirty miles at various compass directions.

Figure 1 is a representation of the NBS/NIST site; the fence line was relocated along the north and east borders during the construction of the access road to southbound I-270 and the widening of Muddy Branch Road in 1986. Within the fence the sampling locations for soil and grass, and the survey markers’ locations are shown. On the fence are marked the original field monitor stations and the current locations selected for four quadrants along the fence line.

## 2. Radioactivity Measurements in Samples

### 2.1 Samples

Beginning in 1961, soil and water samples were taken from the site and the surrounding area. Up to six surface water samples have been sampled in the routine program; currently four samples are taken—one from the stream leaving the NIST site at the western boundary and three from streams nearby. Many wells existed in 1961; as many as 28 well samples were taken at one point in time. The introduction of piped water supplies caused many of the wells to shut down. One well is routinely sampled currently. Samples were taken monthly for a number of years; the current schedule employs a quarterly sampling routine.

Soil samples were taken in late 1961 near the locations of the five surveyors’ marker plaques installed by the US Geological Survey. Soil samples are taken during the dormant vegetation seasons; currently, the sampling schedule is monthly during October through March, the dormant seasons.

Vegetation has been taken since the early 1960s. Before the grounds grooming began, wild vegetation from the fallow fields was gathered. Currently, four grass samples are taken monthly from April through September. For a number of years, from about 1965 through 1985, four onsite plots were roped off and reserved for health physics weekly mowing, yielding a composite sample for analysis.

### 2.2 Analyses

Water and liquids extracted from samples, especially grasses, have been measured with liquid scintillation. Radiochemical processing, using published techniques [3], was employed to extract specific radionuclides until 1984. Gamma spectroscopy using NaI(Tl), GeLi, and InGe systems has been used from the first. In 1984 gamma spectroscopy replaced radiochemical processing as the prime method for analyzing samples for effluents.

Nuclides specifically sought at various times included ^3^H, ^60^Co, ^90^Sr, ^131^I, ^137^Cs, ^222^Rn, ^226^Ra, and ^nat^U. Gamma spectroscopy measurements would have shown any other gamma emitters of sufficient energy and intensity to trigger notification. Gross counting with internal gas flow proportional counters for alpha and beta emitters would have shown those, had there been sufficient activities.

To date, no nuclide originating from NBS/NIST operations has been detected through this routine sampling program. Nuclear weapons fallout activity has been identified—shown to be fallout by examination of location of the sample taken and potential release points for agency processes and determinations that there was no relation between the two. Activities determined to be fallout were found in off site samples, as well as in on site samples. Natural radioactivity has been identified; ground water has a substantial radon content, relative to surface water. For many years, the standard measurement technique for monitoring gamma rays in a large volume water sample was to gather a large volume of water and introduce 30 L (8 gal) into a tank that had a 7.5 cm diameter by 7.5 cm length (3 in × 3 in) NaI(Tl) detector centered in the tank. Following a measurement, the water was drawn off and agitated to remove gaseous products. The gaseous activity levels in ground water, i.e., radon, were reduced with this agitation by a factor of about 10, to the levels of surface water.

## 3. External Radiation Field Measurements

Measurements of the external radiation fields were made using the instrumentation and equipment available, with resulting recording of data in the units in use at the time. For this presentation, those units, usually in exposure terms, i.e., mr or mr per unit time (now termed mR or mR per unit time), have been converted to air kerma rate units, i.e., nGy/h, by evaluating the period of the measurement, if necessary, and introducing a time factor. Where an uncertainty is indicated, the value shown is the combined standard uncertainty (i.e., estimated standard deviation) based on a sequence of measurements or a series of measurement sequences over the time period indicated, e.g., 1 year. The techniques used to quantify the external ionizing radiation fields are described below.

### 3.1 Scintillation Survey Meter

An instrument designed for uranium prospecting^1^,^2^ was used to take measurements in and around the new site in 1960. The sensing element was a 7.5 cm diameter by 7.5 cm long (3 in × 3 in) NaI(Tl) crystal. Calibration was customarily done with ^60^Co, although no calibration data was recorded with the measurements. Full scale indicators for its five ranges were marked from “0.025 mr/h” to “100 mr/h”. Table 1 shows the data recorded with this instrument.

### 3.2 Vibrating Reed Measurements

A vibrating reed ion chamber instrument normally used as a laboratory device^3^ was fitted with a gasoline powered generator and taken to the site for measurements. Calibrations were made with exposures to ^60^Co, both in the laboratory and in the field. Several measurements were made but the difficulties encountered with the power source were, finally, determined to be insurmountable, so its use was discontinued. Table 2 shows the few results obtained.

### 3.3 Film Dosimetry

From 1965 through early 1969, a commercial film badge processor supplied a monthly set of 60 film badges that were placed around the perimeter of the site. Three of those badges were kept at locations away from the site and used as reference monitors; the background used by the supplier to obtain net results for the set was based on the response of an ion chamber at the supplier’s distribution center, in California. The data from the program was only selectively preserved; the recorded data on file show only one set from 1966 and one from 1969, with full years of information for 1967 and 1968, although full term monitoring for all the years is implied in internal publications [2]. The sole recorded data set on file for 1966 shows no start date so the period is unknown. Table 3 shows the information from the recorded data sets, with the period for the single 1966 data assumed the same as for the first period in 1967. The data shown are converted from dose in mrad to air kerma rate in nGy/h, without correction factors [4] applied. No negative values were reported by the film processor. One location’s result in the 1966 data set indicated 72 mrad gamma for the monitoring period; other data within that set exhibited extremely wide variations. Film dosimeters are known to be notoriously susceptible to various environmental effects such as temperature and humidity, and since the data show some extreme variations that are unexplained, the decision to replace film with thermoluminescent dosimetry for perimeter monitoring was not difficult. These data are obviously of little benefit in determining radiation exposure levels at the Gaithersburg site.

### 3.4 Shonka Vibrating Fiber Tissue-Equivalent Ion Chamber

In 1964, Dr. Francis R. Shonka generously provided a prototype of his tissue equivalent ion chamber and electronics [5] to test as an environmental monitor at the new NBS site. The instrument was readout by viewing a conducting fiber through a microscope. The fiber vibrated between binants according to the magnitude of an alternating current field imposed on the binants. The term “binant” was used by Dr. Shonka to indicate separated, hollow, hemispherical elements. Adding a bucking DC voltage to the binants to offset the signal induced on the fiber by the ionization produced in the coupled ion chamber and measuring the time between maximum deflection and null gave an indication of the ionizing radiation field strength. Calibrations were made with exposures to ^60^Co.

The first chamber was pseudospherical, formed by welding strips of plastic with a radiation response approximately equivalent to that of human muscle tissue end-to-end and side-to-side, so a series of flats approximated a spherical shape. The gas filling the chamber was also tissue equivalent. The first electronics were rudimentary, and extremely sensitive to operator control. In 1966, continuing and increasing instabilities of the instrument had become so great that measurements were no longer reliable. A commercial version^4^ of the instrument, with a true spherical shape for the ion chamber and more stable and easily controlled electronics, was procured and placed in service. This version served until the termination of the program in 1973.

From the first, it was seen that the instrument was very sensitive to weather and other conditions. A wind of any magnitude could cause instability in the fiber pattern, possibly because the detector sphere had over 30 cm diameter and wind would induce vibrational motions in the unit. The black color of the sphere caused a response to temperature; summer temperatures required that time be spent so the ball temperature could stabilize, and any changes in volume or pressure could not be immediately determined and accounted for. Umbrellas were occasionally used to provide shade for the instrument when measurements were made in bright sunlight. Extreme caution had to be taken to ensure an adequate electronic grounding point. At first, a large screwdriver was carried from location to location and driven into the ground with water poured on the tool; eventually, copper rods were driven into the soil beside the surveyors’ markers for permanent grounding couplers.

Although the data from these measurements were very informative, a combination of factors forced a decision to terminate the program. The primary influence was the effort required to maintain stability for the instrument in field measurements and the labor required to generate data. Since thermoluminescent dosimetry was seen as the future mechanism that would be acceptable for field measurements, the decision was made to terminate the Shonka project.

**Fig. 2:**
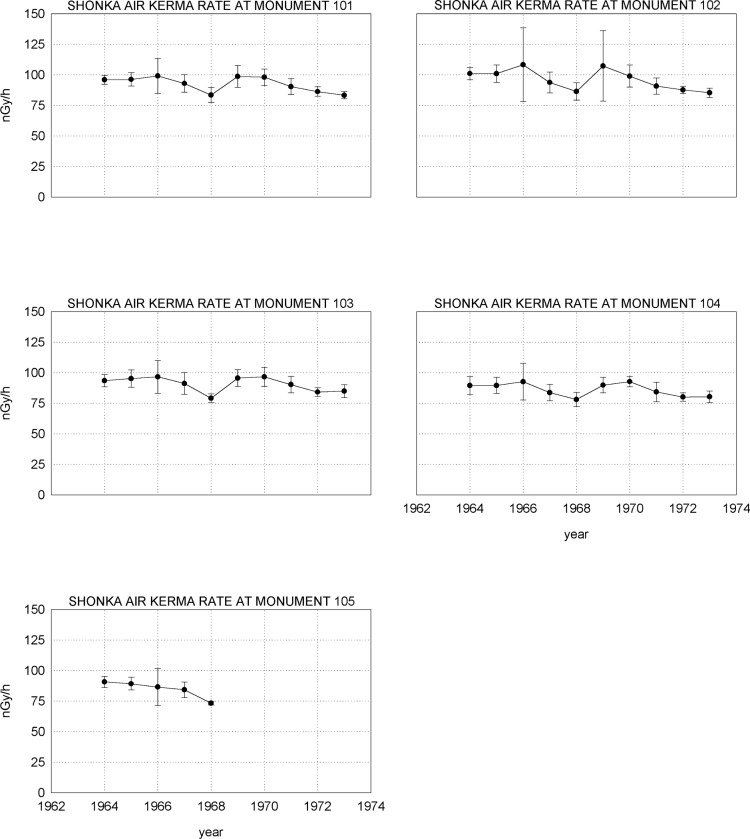
Responses of the Shonka ion chamber at the indicated surveyors’ marker sites on the NBS campus. The variation indicated is the combined standard uncertainty from the measurements within the year.

Table 4 gives the basic evaluations of the data derived from the program. Figure 2 is a graphical representation of the measurement results for each year, with the combined standard uncertainty indicated. The data are in air kerma rate units, since the calibration and field responses were recorded using exposure units, even though the chamber was tissue equivalent and its response would more appropriately be observed in absorbed dose units. The standard deviations shown are just the sample standard deviations for the total set of measurements at a location for each year.

Obviously, from the number of measurements shown for each location, the data do not represent a weekly visit to each and every site. Locations 101 and 102 were visited almost every time. Table 5 shows the number of weekly visits during each month of each year of the project. For location 105, construction of Building 233 precluded measurements after 1968. Toward the end of 1965, the original unit, particularly the electronic control package, began experiencing problems that we could not solve. It was replaced in early 1966 with a commercial package and detector. Setup and calibrations took up most of the first part of 1966, so measurements were not made until July.

In 1967, in June and again in July, a colleague from the Armed Forces Radiobiological Research Institute brought four stray radiation chambers^5^ and made measurements alongside the Shonka chamber at marker 101. Taking measurements over three to four hours, the air kerma rate result from those was 77.6 +/− 6.4 nGy/h in the June trial; in July, the result was 79.6 +/− 16.6 nGy/h. For comparison, the Shonka results, obtained in six trials of approximately one minute each, yielded 96.7 +/− 2.0 nGy/h in June and 101.2 +/− 3.2 nGy/h in July.

### 3.5 High Pressure Ion Chamber

In 1990, a high-pressure ion chamber^6^ (HPIC) was taken to each of the surveyors’ markers and a measurement of at least 24 h was made. Calibrations were made with exposures to ^60^Co. Table 6 shows the results of those tests.

### 3.6 Thermoluminescent Dosimetry

**Fig. 3:**
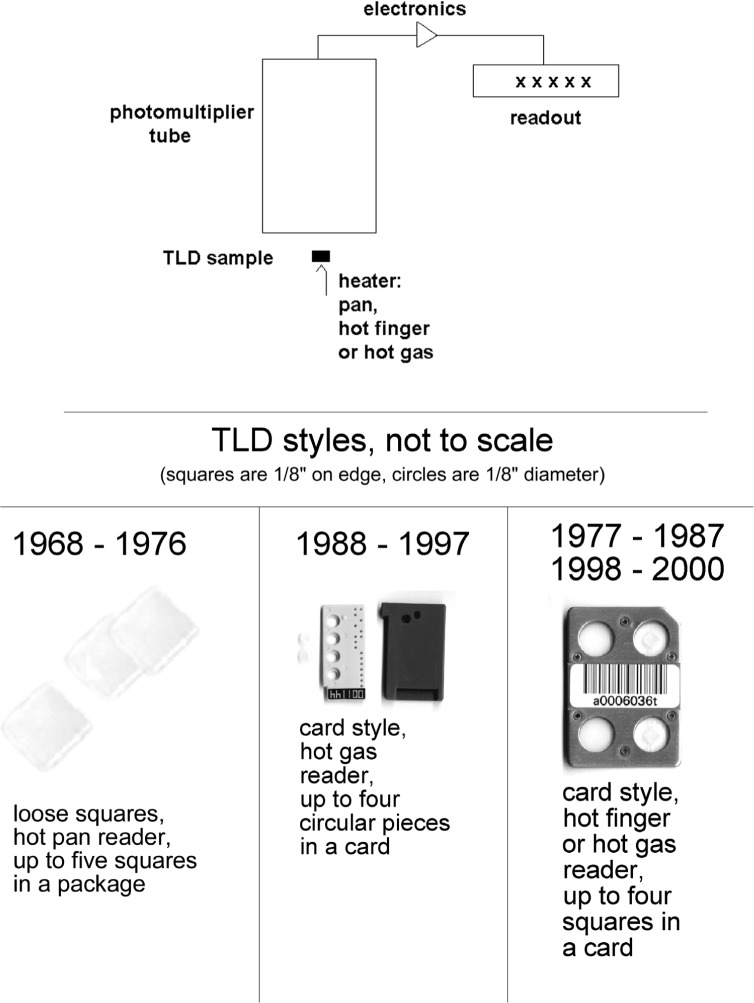
A schematic diagram of the basic elements of the TLD system and pictures of the various types of TLD monitor devices used.

Investigations on thermoluminescent dosimetry (TLD) for environmental monitoring began about 1962. By about 1967, the technique had progressed sufficiently that TLD was introduced into the NBS program, and has been employed since as the primary demonstrator of radioactive effluent control from NBS/NIST operations. Figure 3 shows the basic instrumentation and several types of the monitor devices that have been used in the program. TLD is nearly universally recognized as suitable for this purpose, and the results are accepted by regulatory authorities if a reasonable quality assurance and quality control program associated with environmental monitoring is in place. Some investigations were also made at the start of this program into the use of radiophotoluminescent glass, but results never gave the sensitivity, precision, stability, nor the ease of use determined to be necessary. The details of the TLD techniques are given below.

#### 3.6.1 Materials

A variety of materials have been subjected to test. Configurations include powders, TEFLON^7^ impregnated with LiF, square and round rods, and, finally, the selected configuration, thin squares of extruded melted LiF and CaF_2_ that have selected, precisely controlled impurities, e.g., Mn or Mg^8^. The squares were initially 0.64 cm (1/4 in) on a side, but were soon replaced with a higher sensitivity square of 0.32 cm (1/8 in) on a side. CaF_2_ was used in some monitor devices, but the mainstay and the currently used material is LiF. Compared to LiF, CaF_2_ exhibits a markedly greater sensitivity, but also has a response to photon radiations that is less similar to tissue, and has a greater fading characteristic, very noticeable when used over an extended period.

Loose squares were annealed according to the accepted protocols, including a high-temperature anneal at 400 °C, followed by a prescribed cool-down period and a lower temperature annealing. Eventually, it was found that the readout heating was sufficient to erase the signal in the material so that it could be reused without the high-temperature annealing, given the low exposures received by the material at environmental levels.

Eventually, a system was introduced that used squares permanently encased in transparent plastic covers. The plastic covers on the squares in the cards would not withstand the high temperature anneal, so these card-type devices are used according to manufacturer’s recommendations, i.e., program use with only reader annealing for low exposures. The readout cycle seems to provide adequate heating for signal erasure and that mechanism has been used since the introduction of the card-type devices for the program.

#### 3.6.2 Packaging and Handling

Many packaging configurations were tested, before and after selecting the method finally used. From 1968 until 1975, loose, individual squares of TLD were positioned on a cardboard or a thin fiberboard backing, wrapped in plastic film, and taped with black electrical tape. These squares were, from early in the program, handled with a vacuum pickup tool that helped eliminate any introduction of foreign matter, e.g., finger grease, and reduce triboluminescence caused by tweezer manipulation. In 1975, an automated system was introduced, using cards that held square TLD ribbons encased in transparent plastic covers that would withstand the hot finger used for generating the signal. Since then, the TLD materials have been incorporated into card formats and automatically manipulated. Cards are placed in coin envelopes, and these, in turn, are placed in plastic sleeves, either with a zipper-type closure or heat sealed.

The loose square packaging proved stable, but also proved to attract birds. More than a few of the packets on the fence were attacked and destroyed by birds pecking at the packet. After some years, mini-size electrical boxes were fitted with hose clamps to attach to fence posts and the packets were placed in these for protection. Currently, plastic spring-loaded, hinged-lid boxes designed for outdoor electrical receptacles, with hose clamps riveted to the back plate, are used for packet holders.

#### 3.6.3 Reader Systems

The initial readout device^9^ had a relatively massive heater pan for holding the TLD square for readout, so it took a considerable time to cool before a second reading could be made. The display was analog and, thus, susceptible to operator interpretation error. Very shortly it was replaced with another instrument^10^ that had a much less massive pan so it cooled much more quickly. It also had an analog display; that was replaced with a digital display before it was placed in service. Eventually, the electronics were bypassed and the photomultiplier signal was sent to an electrometer and separate digital display for signal processing. This served until 1975, when an automated system^11^ was introduced that used TLD squares packaged in sealed cards. In 1988, this was, itself, replaced with another instrument^12^ that used an automated vacuum needle to lift the squares from their holders into a heated gas stream for readout. In turn, this was replaced with a card with squares sealed in place and a hot gas readout system. This system^13^ has been in use since 1998.

#### 3.6.4 Procedures

From the program’s initiation in 1968 until 1973, three LiF squares were used; then, for a few years, three LiF and two CaF_2_ squares were used. Eventually, until the automated system was acquired in 1975, two CaF_2_ squares were used. Light and liquid impervious packets enclosing the squares were prepared and exchanged on a monthly basis. The individual squares were annealed each time, just prior to use, and packaged for distribution.

The first cards used with the automated system were packaged so a card with two LiF squares and a card with two CaF_2_ squares were put together and distributed on a monthly basis. In 1982, the CaF_2_ card was eliminated, and a quarterly exchange period was begun, using a single card with two squares of LiF. The hot-gas reader was introduced in 1988 with an increase to four squares of LiF in each card holder. With the current system, three LiF squares are used in each card.

#### 3.6.5 Measurement Results

**Fig. 4:**
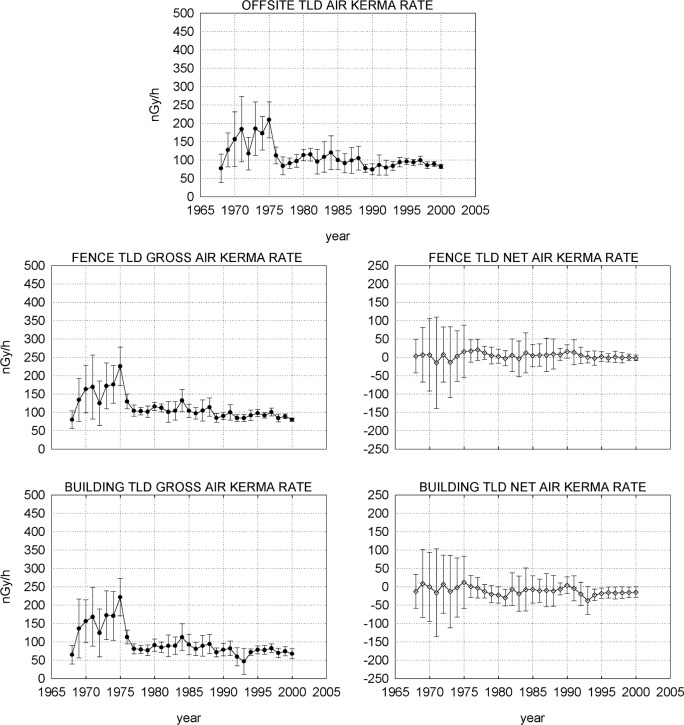
Responses of the TLD monitors at offsite locations, on the NBS/NIST fence, and atop buildings on site. The offsite results are used as background measurements for deriving the net results for all other results. The variation indicated is the combined standard uncertainty from the measurements within the year.

Figure 4 shows the results of the offsite measurements, the results of measurements atop the buildings, and the total fence line measurement results, with the combined standard uncertainty indicated. The offsite results are used as background values for the NIST onsite measurements. Subtraction of the offsite results from the various NIST results yields net curves for the NIST data.

**Fig. 5:**
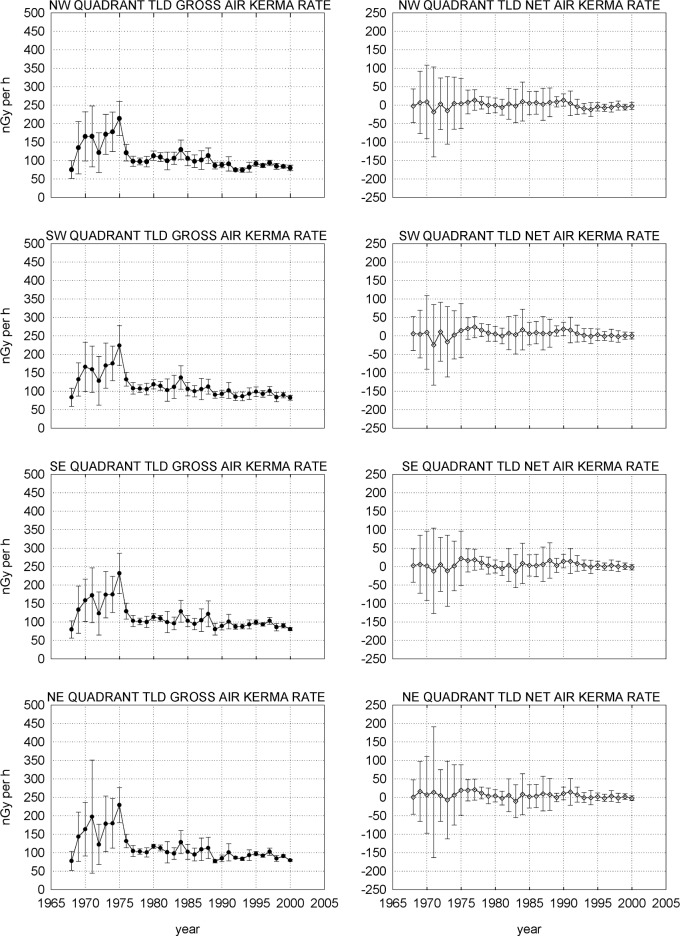
Responses of the TLD monitors on the NBS/NIST fence, with locations grouped by quadrants. The variation indicated is the combined standard uncertainty from the measurements within the year.

Figure 5 shows the results of monitoring in the four quadrants of the fence line, using the mid-point of the line from the Reactor stack to the Linac stack as a grid center. For the data taken up to 1988, the southwest and southeast quadrants obviously had more data points than the other two quadrants. For the data after 1982, when the monthly exchange procedure was changed to a quarterly exchange procedure, each quadrant has four locations supplying data. The standard uncertainty is indicated.

Table 7 shows the times and changes in the TLD protocol for the NBS/NIST environmental radiation field monitoring program. From this information, certain observations might be drawn concerning the seemingly erratic behavior of the data displayed in the graph.

Note, first, that the curve shape for the gross data is the same for all groupings of TLD responses, whether offsite, on buildings, for the total fence set, or for any of the quadrant sets. Second, note that all the net data, i.e., the gross data with background removed, is at or near the zero line, indicating that there has been no measurable effect from any radiation field contributor on the fence or on the buildings that isn’t observed in the offsite, or background, data, as well.

In the first few years, the error bars are significantly out of line with the following years. This seems to correlate with the manual handling and manipulation of the individual squares of TLD, even though vacuum pickup tools were used to manipulate the squares for most of the time, observed when compared to data from the automated systems. The variations may well have been due to the phosphor, the process, the system, or a combination of these three factors that were used for the measurements at that time. It is now known that the manual handling of the TLD squares introduces mechanical stress, causing a variation in the result of a measurement [6,7]. One researcher tells of a scientist manipulating solid pieces of thermoluminescent material with tweezers when data anomalies were noted. Eventually, a correlation was made between the occurrence of an anomaly with the ringing of the telephone in the laboratory. Apparently the noise caused an inadvertent twitch with increased pressure on the tweezer handles.

There obviously was some bias, unexplained to date, in the process in that early period that contributed to the shape of the response curve, as opposed to the relatively flat shape in subsequent years. Of course, when the background is removed, that portion of the curve, as seen in the net values, although with substantial uncertainties, tends to fall on or near the zero line, very similar to the data from the later periods.

Attempts to normalize the early data using values from an internal instrumental light source and two scintillating buttons with tritium-impregnated polystyrene that were measured with each set of devices, and values from the squares exposed to known levels of ^60^Co or ^137^Cs did not change the shape nor the amplitude of the gross or the net curves for any set of data. Examination of averages and median values for the net data sets showed that in all cases there were nearly equal numbers of values above and below these central points.

Noting the increases in uncertainties in the early 1980s, it can be noted that the material changed from CaF_2_ to LiF. As previously noted, CaF_2_ has a significantly higher response to photons than does LiF, so exposure to a given radiation field would give a significantly greater signal for CaF_2_ than for LiF. Simple statistics will give a lower uncertainty for a larger signal from the same origin; this is the presumed reason for the magnitude of the differences in uncertainty.

## 4. Other Measurement Techniques

A number of other techniques and measurement systems have been tried and rejected and the search continues for any measurement mechanism that would offer advantages for routine environmental monitoring and sampling.

Among the current tests and investigations, the electret ion chamber radiation detector^14^ and a geiger tube (G-M) monitor with a variable count accumulation time^15^ have shown much promise. The electret device is a 50 mL conducting plastic chamber with a charged Teflon disk serving as an ion collector. Sealed in a protective envelope against radon penetration, the device in tests has demonstrated equivalent sensitivity and precision to the TLD device [8,9]. The G-M device has a programmable integration time so sudden changes in field intensity can be observed. It also seems to have characteristics equivalent to TLD integrators.

One instrument^16^ recently used at NIST has a detector and potential uses that might make it tempting to view instrumentation as having come full circle from the first attempt, i.e., a NaI(Tl) detector with a possible use as a prospecting tool. The current device, however, has relatively sophisticated electronics for spectral accumulation and for data transmission, and a software program that permits extraction of extremely small specific energy photon signals from a full spectrum. One test showed a spike from ^41^Ar, the 1.8 h half life gas in the reactor stack effluent, even though the magnitude of the spike was many orders of magnitude less than the magnitude of the natural background. While not a part of the routine monitoring program, this instrument should prove useful for specific investigations in the future.

## 5. Conclusions

Measurements begun well before the occupancy of the NBS/NIST Gaithersburg laboratories and continuing through the 40 years following the first measurements clearly indicate that no effluents, emissions, fields, or other radiological products from agency operations have impacted the environment. Data involved are from the sampling programs for water, grass, and soil, and the external field investigations, using both tissue-equivalent and high pressure ion chambers, survey meters, and TLD. No positive value from the routine measurement program, other than those determined to be from natural sources or from fallout, has been observed.

## Figures and Tables

**Table 1:** Scintillometer survey meter data

Meas. date	Location	Air kerma rate (nGy/h)
June 8, 1960	Marker #102	78.8
	Marker #103	96.4
	Marker #104	83.2
June 15, 1960	NE corner, Brooks & Russell Avenues	70.1
	NE corner, Brooks & Summit Avenues	70.1
	NE corner, Frederick & Summit Avenues	75.3
	SW corner, railroad & Summit Avenue	74.5
	Park Ave. in front of N. laundromat	50.1
	SE corner, Russell & E. Diamond Avenues	49.9
	Marker #101	74.5
Sept. 16, 1960	Marker #102	87.6
	Marker #103	78.8
	Marker #104	52.6
	Marker #105	52.6
	NE corner, Browns Station & Clopper Roads	65.7
	Browns Station Rd. under I-70S overpass	74.5
	Opposite Browns Station Rd. at Rte 355	100.7
	NE corner, Maryland Ave. & Rte. 355	56.9
	SE corner, Rte. 355 & Brooks Ave.	35.0
	NE corner, Rte 124 & Rte. 355 (RR bridge)	56.9
	S corner, Brooks & Summit Aves.	74.5
	SW corner, railroad & Summit Ave.	83.2
	NE corner, Summit Ave. & Rte. 355	56.9
	SW corner, Muddy Branch Rd. & Rte. 124	52.6
	S of Rte. 124 at city limit sign	74.5
	SW corner, Rte. 124 & NBS entrance	78.8

**Table 2:** Vibrating reed electrometer data

Meas. date	Location	Air kerma rate, with 1 s.d. (nGy/h)
June 6, 1960	Marker #104	61.6 +/−12.1
Sept. 16, 1960	Marker #104	91.7 +/− 20.9
Aug. 15, 1962	Marker #104	127.8 +/− 66.1
Sept. 10, 1962	Marker #101	139.8 +/− 36.8

**Table 3:** Film air kerma rate (nGy/h)

Location	1966(1 data set)	1967	1968	1969(1 data set)
Offsite	70.2	13.6	9.3	0.0
Fence	195.3	14.3	4.9	0.0

**Table 4:** Air kerma rate in nGy/h units and number of measurements for each year from Shonka vibrating fiber electrometer

Location and data set	Average values of air kerma rate and data for the year									
	1964	1965	1966	1967	1968	1969	1970	1971	1972	1973
Location 101										
Average value	95.9	96.2	99.0	93.0	83.5	98.8	98.1	90.4	86.3	83.3
Standard deviation	3.6	5.6	14.3	7.2	6.2	9.0	6.7	6.4	3.8	2.9
Median value	95.9	96.7	94.4	93.3	84.2	98.2	98.1	90.3	86.4	83.1
Maximum value	100.4	115.7	137.3	111.3	94.7	135.0	110.7	103.5	94.1	90.2
Minimum value	88.4	82.8	82.3	75.7	71.1	83.6	86.3	78.5	80.0	77.8
No. measurements	12	54	53	114	51	187	50	70	25	70
Location 102										
Average value	101.1	101.0	108.3	93.8	86.4	107.3	98.9	90.8	87.7	85.3
Standard deviation	5.0	7.2	30.2	8.5	7.1	28.9	9.1	6.8	2.9	3.9
Median value	104.2	100.4	96.9	95.5	86.5	98.5	99.1	91.1	87.9	84.9
Maximum value	105.6	127.9	209.8	109.5	100.9	245.5	117.9	102.3	91.5	93.4
Minimum value	90.1	86.7	86.7	70.5	74.9	86.3	83.3	76.6	82.3	76.3
No. measurements	9	56	57	108	51	232	50	70	25	71
Location 103										
Average value	93.5	95.2	96.6	91.2	79.1	95.5	96.5	90.3	84.2	84.9
Standard deviation	5.0	7.0	13.4	8.9	3.5	6.9	7.5	6.8	3.6	5.3
Median value	93.5	93.7	91.5	91.9	78.1	94.1	94.1	89.6	83.9	84.6
Maximum value	100.4	121.2	131.6	116.4	85.3	120.0	113.9	103.5	92.2	99.6
Minimum value	86.9	84.4	76.5	66.3	74.4	86.5	85.7	78.7	77.3	73.0
No. measurements	9	51	56	102	30	70	50	70	27	75
Location 104										
Average value	89.5	89.6	92.7	83.7	78.1	89.8	92.8	84.3	80.1	80.3
Standard deviation	7.5	6.5	15.0	6.7	5.9	6.3	4.3	8.0	3.5	4.7
Median value	87.8	88.7	87.2	83.3	76.0	87.9	92.6	83.3	79.5	80.0
Maximum value	102.3	109.3	129.1	106.3	92.5	107.8	103.8	101.9	87.0	93.1
Minimum value	82.5	76.6	76.4	67.4	71.8	82.1	84.4	70.5	73.7	70.3
No. measurements	6	46	57	108	30	60	40	55	25	70
Location 105										
Average value	90.7	89.2	86.5	84.3	73.2	None	None	None	None	None
Standard deviation	4.6	5.2	15.2	6.4	1.5					
Median value	91.9	88.3	80.4	85.0	73.5					
Maximum value	96.2	106.1	134.3	98.7	74.8					
Minimum value	83.2	76.1	72.0	69.4	70.5					
No. measurements	9	45	55	84	6					

**Table 5:** Schedule of visits to the various locations

	Number of weekly visits in each month in each year to one or more of the locations									
Month of meas. set	1964	1965	1966	1967	1968	1969	1970	1971	1972	1973
Jan		1		1	1					
Feb		1		1	2					
Mar					1				1	1
Apr		1			1			2	1	1
May	1	3		2		2	2	1		1
Jun		3		3	1	2	2			1
Jul		5	1	2		3	1			1
Aug		4	1	2		2		1		
Sep		1		1	1	2		1		1
Oct	1		3	3		2				1
Nov	2	1	2	2		1		1		
Dec	2		1	1		1				

**Table 6:** High pressure ion chamber data

Meas. period	Location	Air kerma rate and 1 s.d. (nGy/h)
Continuing meas. From Sep. 14, 1990 through Nov. 1, 1990	Marker #101	99.2 +/− 3.1
	Marker #102	102.1 +/− 3.2
	Marker #103	95.2 +/− 3.0
	Marker #104	87.4 +/− 2.7
	Marker #105	96.6 +/− 3.0

**Table 7:** TLD Process Event Calendar

Period	Event
1968–1988	60 TLD devices distributed (34 to 38 on fence)
1968–1981	Monthly exchange of TLD devices
1968-late 1976	Manual manipulation of individual squares of TL material
1968–1972	Three squares of LiF
1973–1975	Three squares of LiF and two squares of CaF_2_
1976	Two squares of CaF_2_
1977–1987	Automated card with enclosed and contained TL squares, hot finger
1977–1981	Four squares of CaF_2_
1982–1987	Two squares of LiF
1982–2000	Quarterly exchange of TLD devices
1986	Relocated north and east fence line for highway construction
1988–1997	Automated card with three LiF squares, lifted automatically from holder
1989–2000	25 to 27 TLD devices distributed (16 on fence)
1998–2000	Automated card with three enclosed and contained LiF squares, hot gas

## Footnotes

1 Certain commercial equipment, instruments, or materials are identified in this paper to foster understanding. Such identification does not imply recommendation or endorsement by the National Institute of Standards and Technology, nor does it imply that the materials or equipment identified are necessarily the best available for the purpose.

2 20th Century Electronics, Canada, Mark IV Scintillometer.

3 Applied Physics Corp., Cary Model 31.

4 Emdeco Corp., Shonka Control Unit, Model 1.

5 STRAD, various models of large-volume condenser ion chambers, Victoreen Instrument Co.

6 Reuter-Stokes, model RSS-111.

7 TEFLON is a registered trademark of DuPont.

8 Harshaw Chemical Co., high-sensitivity TLD materials.

9 Radiation Detection Co., model 1100.

10 Eberline Instrument Co., model TLR-5.

11 Harshaw model 2271.

12 Alnor Instrument Co., model DOSACUS.

13 Harshaw model 6600.

14 Rad Elec Inc., e-perm environmental monitor.

15 Genitron Instruments GmbH, GammaTRACER.

16 Exploranium Radiation Detection Systems, GR-320.
